# Mortality risk for kidney transplant candidates with diabetes: a population cohort study

**DOI:** 10.1007/s00125-024-06245-x

**Published:** 2024-08-06

**Authors:** Raja Rashid, Daoud Chaudhry, Felicity Evison, Adnan Sharif

**Affiliations:** 1 Department of Nephrology and Transplantation, University Hospitals Birmingham, Birmingham, UK; 2 University Hospital of North Midlands, Stoke on Trent, UK; 3 Department of Health Informatics, University Hospitals Birmingham, Birmingham, UK; 4 Institute of Immunology and Immunotherapy, University of Birmingham, Birmingham, UK

**Keywords:** Diabetes, Dialysis, Kidney failure, Kidney transplant, Mortality

## Abstract

**Aims/hypothesis:**

It is unclear whether kidney transplant candidates with diabetes have equitable transplantation opportunities or have divergent survival probabilities stratified by kidney replacement therapy. The aim of this study was to investigate these two issues using national transplant registry data in the UK.

**Methods:**

A cohort study was undertaken of prospectively collected registry data of all wait-listed people with kidney failure receiving dialysis in the UK. All people listed for their first kidney-alone transplant between 2000 and 2019 were included. Stratification was done for cause of kidney failure. Primary outcome was all-cause mortality. Time-to-death from listing was analysed using adjusted non-proportional hazard Cox regression models, with transplantation handled as a time-dependent covariate.

**Results:**

A total of 47,917 wait-listed people with kidney failure formed the total study cohort, of whom 6594 (13.8%) had diabetes classified as cause of kidney failure. People with kidney failure with diabetes comprised 27.6% of the cohort (*n*=3681/13,359) that did not proceed to transplantation vs only 8.4% (*n*=2913/34,558) of the cohort that received a transplant (*p*<0.001). Kidney transplant candidates with diabetes were more likely to be older, of male sex and of ethnic minority background compared with those without diabetes. In an adjusted analysis, compared with remaining on dialysis, any kidney transplant provided survival benefit for wait-listed kidney transplant candidates regardless of diabetes as cause of kidney failure (RR 0.26 [95% CI 0.25, 0.27], *p*<0.001).

**Conclusions/interpretation:**

Kidney transplant candidates with diabetes have a lower chance of transplantation despite better survival after kidney transplantation vs remaining on dialysis. The reasons for this require further investigation to ensure equal transplantation opportunities.

**Graphical Abstract:**

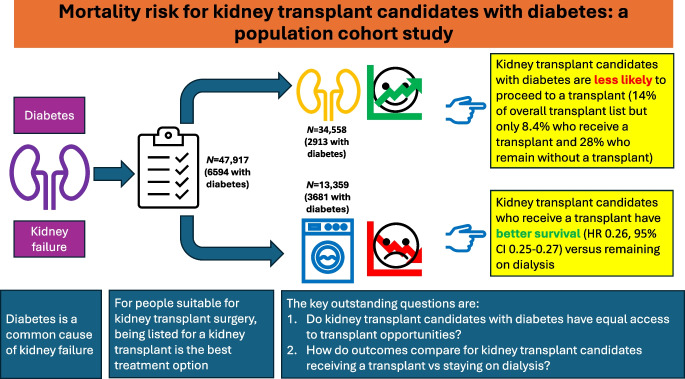

**Supplementary Information:**

The online version contains peer-reviewed but unedited supplementary material available at 10.1007/s00125-024-06245-x.


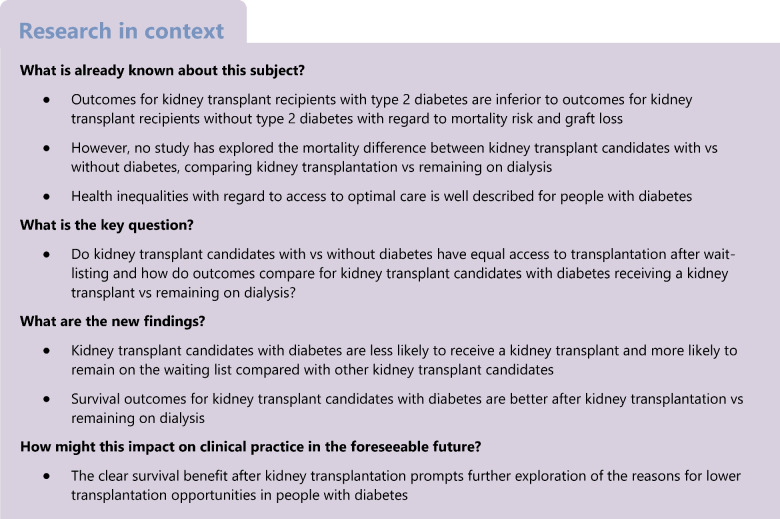


## Introduction

Diabetes remains a significant public health issue. According to the Global Burden of Disease Study 2021, 529 million people are living with diabetes globally and this is projected to increase to 1.32 billion by 2050 [1]. With regards to complications, diabetes remains the leading cause of kidney failure in many countries across the world according to national registry reports. For people living with kidney failure who are fit enough to be listed for transplant surgery, kidney transplantation is the best treatment for end-stage kidney failure and improves long-term survival vs remaining on dialysis [2].

However, two important issues have not been explored. First, many studies demonstrate inferior long-term outcomes after kidney transplantation for people living with vs without diabetes [3–5]. While important for awareness and counselling, this difference is irrelevant for clinical decision making. For people with kidney failure who have diabetes, an assessment of their long-term outcomes after transplantation should not be compared against people without diabetes but against people living with kidney failure who have diabetes and are suitable candidates but do not receive a kidney transplant. Second, there is evidence that some people living with diabetes have inequity of access to the best treatments [6], which may extend to kidney replacement therapy. For example, according to the 2021 UK Renal Registry report, 31.3% of people starting kidney replacement therapy have diabetes listed as a comorbidity [7]. However, according to UK Transplant Registry data, only 20.0% of adult registrants on the active kidney-only transplant list between 2022 and 2023 had diabetes listed as their primary renal disease [8]. This may be due to different registries using different classifications, with the inability to differentiate diabetes as a concomitant medical comorbidity vs primary cause of kidney failure. However, it may also suggest inequity of access for people with kidney failure with diabetes when it comes to opportunities for kidney transplantation. The aim of this study was to investigate these two issues using national transplant registry data in the UK.

## Methods

### Study cohort

A cohort study was undertaken of prospectively collected transplant registry data related to all people with kidney failure receiving dialysis in the UK who were added to the national transplant waiting list. All people listed for their first kidney-alone transplant in the UK between 1 January 2000 and 30 September 2019, inclusive, were included in the study. No formal sample size estimate was conducted as all eligible patient records were used and follow-up data was available to 31 December 2020. The study is reported as per STROBE guidance [9].

### Study variables

The following study variables were available for the full study cohort: age (at time of listing and at transplantation if that occurred), sex (as determined by the transplant centre based on biological sex), ethnicity (classified as White, Black, Asian [South Asian], Other, unknown, as determined by the transplant centre based upon self-reported ethnicity), primary cause of kidney failure (classified as diabetes, glomerulonephritis, hypertension, other separate, polycystic kidney disease, pyelonephritis/reflux nephropathy, unknown/missing), year of listing and waiting time. No further information was recorded in the transplant registry regarding the diagnosis of diabetes (e.g. type 1 vs type 2, length of diagnosis, treatment, etc.).

For people with kidney failure who received a kidney transplant, we had a variety of donor- (e.g. cold ischaemia time [CIT], human leucocyte mismatch [HLA] level, calculated reaction frequency), recipient- and transplant-related variables that facilitated subanalyses of interest (e.g. rejection episodes, graft function, death-censored graft survival).

### Study definitions

All types of kidney transplant allografts were included and were stratified into those from living donors or from any deceased donor (inclusive of donors after brain or circulatory death). Deceased donors were further stratified into standard-criteria donors (SCDs) or expanded-criteria donors (ECDs). An ECD was defined by any deceased donor aged ≥60 years or a deceased donor aged between 50 and 59 years with two comorbidities among hypertension, death from cerebrovascular accident or terminal serum creatinine levels >133 μmol/l. By default, all other donors not fulfilling this criterion were defined as SCDs. Categorised by historical data from the USA, ECD kidneys are associated with a 70% increased risk of graft failure compared with SCD kidneys (RR 1.70) [10]. Although the kidney donor profile index (KDPI) now provides transplant professionals with more granular information, this basic stratification of SCD vs ECD kidney remains valid for use in countries outside the USA.

CIT is the time from organ procurement to transplantation, a period signified by ischaemia–reperfusion injury. HLA mismatch levels were graded in accordance with National Health Service Blood and Transplant (NHSBT) classification used during this study period and were defined by mismatches at *HLA-A*, *-B* and *-DR*; level 1 (HLA mismatch 0), level 2 (HLA mismatch 0 DR and 0/1 B), level 3 (HLA mismatch 0 DR and 2B, or 1 DR and 0/1 B) and level 4 (1 DR and 2B, or 2 DR). Calculated reaction frequency (cRF), a measure of sensitisation, was used to calculate the percentage of previous 10,000 deceased donors with which we would expect a positive crossmatch (0% cRF is unsensitised and 100% cRF is the highest possible level of sensitisation).

### Outcomes

The primary outcome of interest was all-cause mortality risk. We performed the survival analysis according to the intention-to-treat principle, meaning people were not dropped from the analysis if they were removed from the waiting list or if transplantation subsequently failed. Secondary outcomes explored included graft-related outcomes (rejection, graft function, death-censored graft loss) for kidney transplant recipients only.

### Statistical analysis

For baseline demographics, continuous variables were reported as medians and IQRs and compared between groups using Mann–Whitney tests. Ordinal factors were also compared using Mann–Whitney tests, while nominal factors were analysed using Fisher’s exact tests and *χ*^2^ tests for those with two categories or more than two categories, respectively. Missing data underwent list-wise deletion and complete case analysis was undertaken.

Survival was analysed as time from placement on the waiting list to death, with data censored at loss of follow-up or on 31 December 2020. Time-to-graft-loss models were conducted using survival/censoring-weighted Cox regression models and adjusted for age at listing, sex, ethnicity, cause of kidney failure, year of placement of the waiting list, level of HLA mismatches, CIT (in hours) and calculated reaction frequency.

For mortality risk analyses, unadjusted survival-free probability was analysed by generation of Kaplan–Meier curves. After testing for violations of the proportional hazard assumption, time-to-death was modelled using non-proportional hazard Cox regression models with transplantation handled as a time-dependent covariate. Using this approach, all people contributed data for time at risk (and death if it occurred) to the non-transplant group starting at study entry before some switched and contributed time at risk (and death if it occurs) to the transplant group starting at the time of transplantation (this formed the time-dependent transplant covariate in the model). Mortality risk HRs were computed for the transplant recipients vs individuals on the waiting list. We explored adjusted models factoring for age, sex, ethnicity, cause of kidney failure and year of placement on the waiting list. We also repeated the analysis after exclusion of people whose kidney failure was due to any other cause, leaving a dataset of people with diabetes only. As the HR may change over time for kidney transplant candidates with diabetes, period-specific HRs using piecewise Cox regression models for specific time intervals post-operatively (0–30 days, 31–180 days, 181–365 days, 1–3 years and 3–5 years) are presented.

Additionally, we performed a secondary analysis if any association was observed for death-censored graft loss, as there is a strong independent association with mortality risk within a year of graft loss [11].

All analyses were done using R 4.2.2 (R Foundation for Statistical Computing, Vienna, Austria), with packages including *coxphw* (survival analyses) [12].

### Approvals

From all people undergoing solid organ transplantation in the UK, the NHSBT obtains informed consent for data collection and subsequent approved analyses (for audit, governance or research). Research study proposals are reviewed and approved by the kidney advisory group on behalf of NHSBT before data dissemination. Study investigators had access to anonymised datasets only.

## Results

### Study cohort

**Fig. 1:**
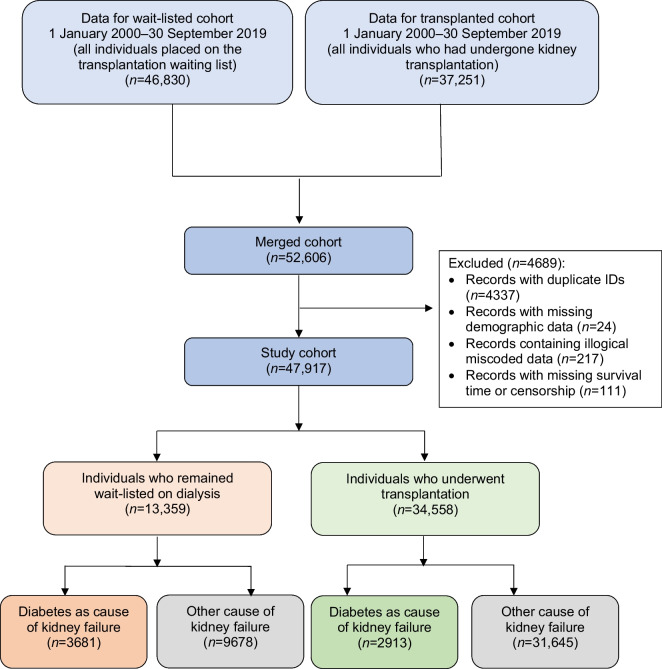
Study cohort flowchart. The original cohort contained records from two datasets: people with kidney failure listed for transplantation (wait-listed cohort) and people with kidney failure who received a kidney transplant (transplanted cohort). Note that some individuals in the wait-listed cohort were also included in the transplanted cohort; duplicated entries were removed from the merged cohort

The original cohort obtained from NHSBT contained records from two datasets between 1 January 2000 and 30 September 2019: people with kidney failure who received a kidney transplant (*n*=37,251); and people with kidney failure listed for transplantation (*n*=46,830). After combining both datasets, duplicated records and/or records with missing demographic data were excluded. This left 47,917 people with kidney failure to form our total study cohort, of whom 34,558 (72.1%) subsequently received their first kidney transplant after wait-listing (living donors, *n*=9140; deceased donors, *n*=25,418). Observation time for the study cohort involved a total of 222,896 person-years, with a median follow-up of 5.8 years. See Fig. 1 for the PRISMA flowchart.

### Baseline demographics

Table 1 highlights significant differences in baseline demographics for wait-listed kidney transplant candidates who receive transplantation vs those that remained without transplantation. Importantly, compared with people with kidney failure without diabetes, a lower proportion of individuals with kidney failure and diabetes were placed on the transplant list (13.8% of the cohort), with more individuals remaining on dialysis. A more detailed breakdown is shown in electronic supplementary material (ESM) Fig. 1 and ESM Fig. 2. We observed that people with kidney failure and diabetes comprised 27.6% of the cohort (*n*=3681/13,359) that did not proceed to transplantation vs only 8.4% (*n*=2913/34,558) of the cohort that received a transplant (*p*<0.001) (ESM Fig. 1). We also noted differences in the type of kidney allograft received: people with kidney failure and diabetes comprised 12.3% of the cohort receiving ECD kidneys (*n*=903/7356) but only 7.5% and 7.2% of the cohort receiving SCD (*n*=1351/18,062) or LD (*n*=659/9140) kidneys, respectively (*p*<0.001) (ESM Fig. 2).

**Table 1:** Baseline demographics of people with kidney failure on the national transplant list

Characteristic	Overall (*N*=47,917)	Wait-listed (*N*=13,359)	Transplant (*N*=34,558)
Age at entry to waiting list, years	49 (38–59)	53 (42–63)	47 (36–57)
Sex			
Male	29,799 (62)	8143 (61)	21,656 (63)
Female	18,118 (38)	5216 (39)	12,902 (37)
Ethnicity			
White	36,239 (76)	9564 (72)	26,675 (77)
Asian	6288 (13)	2072 (16)	4216 (12)
Black	3571 (7.5)	1198 (9.0)	2373 (6.9)
Mixed	145 (0.3)	35 (0.3)	110 (0.3)
Chinese/Oriental	494 (1.0)	155 (1.2)	339 (1.0)
Other	744 (1.6)	226 (1.7)	518 (1.5)
Unknown	436 (0.9)	109 (0.8)	327 (0.9)
Primary renal disease			
Diabetes	6594 (14)	3681 (28)	2913 (8.4)
Glomerulonephritis	2806 (5.9)	511 (3.8)	2295 (6.6)
Hypertension	2505 (5.2)	633 (4.7)	1872 (5.4)
Other Separate	12,418 (26)	2787 (21)	9631 (28)
Polycystic kidney	4636 (9.7)	845 (6.3)	3791 (11)
Pyelonephritis/reflux	3063 (6.4)	592 (4.4)	2471 (7.2)
Unknown/missing	15,895 (33)	4310 (32)	11,585 (34)
Time on transplant list, days	579 (229–1123)	475 (217–858)	638 (234–1231)

Data are expressed as median (IQR) or *n* (%)

We further explored people with kidney failure who were wait-listed (ESM Table 1) or who proceeded with transplantation (ESM Table 2) and compared differences between individuals with vs without diabetes. The wait-listed people with kidney failure and who had diabetes were more likely to be older, of male sex and of ethnic minority background compared with those without diabetes. This was also true for kidney transplant recipients with vs without diabetes, with additional differences seen in baseline immunological variables (see ESM Table 2).

### Unadjusted and adjusted kidney allograft outcomes

We compared kidney allograft outcomes post-transplantation for kidney transplant recipients with vs without diabetes for risk of acute rejection within 1 year, graft function at 1 year (creatinine) and death-censored graft survival. We did not observe any difference in rates of rejection within the first year between kidney transplant recipients with vs without diabetes (10.9% vs 12.6% respectively, *p*=0.212). Neither did we observe any difference in 1 year creatinine (in μmol/l) between kidney transplant recipients with vs without diabetes (141 vs 143 respectively, *p*=0.377). For kidney transplant recipients with diabetes, we observed 566 graft losses (19.4% of *n*=2913) while in kidney transplant recipients without diabetes we observed 6327 graft losses (20.0% of *n*=31,645), which was not significantly different (*p*=0.466).

For death-censored graft survival we undertook a weighted Cox regression model to account for the differences in baseline demographics and immunological variables. In this adjusted analysis, we did observe an association with increased risk for graft loss (HR 1.36 [95% CI 1.20, 1.53], *p*<0.001) for kidney transplant recipients with vs without diabetes (see ESM Table 3).

### Unadjusted and adjusted mortality events

**Fig. 2:**
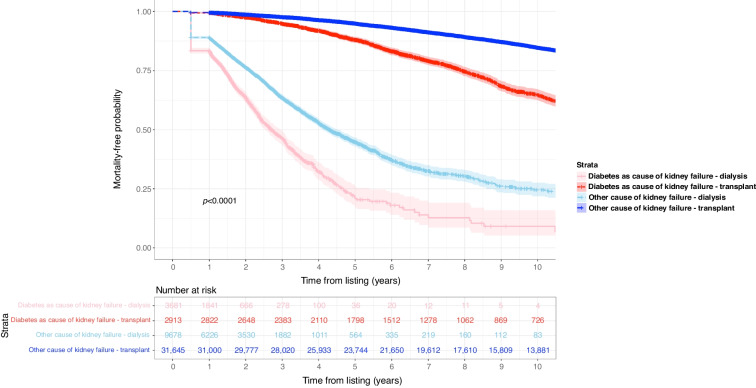
Unadjusted Kaplan–Meir plot of mortality-free survival comparing people with kidney failure with or without diabetes as cause of kidney failure stratified by kidney replacement therapy (dialysis or transplantation)

In the primary study cohort, we observed a total of 10,698 deaths (22.3% of study cohort). Kidney transplant candidates with diabetes as cause of kidney failure had greater mortality risk after being added to the transplant waiting list compared with those without diabetes as cause of kidney failure (31.1% vs 20.9% respectively, *p*<0.001). For people with kidney failure and diabetes, we observed 883 deaths (30.3% of *n*=2913) after kidney transplantation vs 1169 deaths (31.8% of *n*=3681) in those remaining on dialysis. For people with kidney failure without diabetes, we observed 5812 deaths (18.4% of *n*=31,645) after kidney transplantation vs 2834 deaths (29.3% of *n*=9678) in those remaining on dialysis (see Fig. 2).

In a time-dependent non-proportional hazard Cox regression model, compared with remaining on dialysis, any kidney transplant provided survival benefit for wait-listed kidney transplant candidates regardless of diabetes as cause of kidney failure (HR 0.26, 95% CI 0.25–0.27, *p*<0.001) (Table 2). In view of the association between kidney transplant recipients with diabetes and graft loss, we performed a second analysis to account for the risk of graft loss. In a time-dependent non-proportional hazard Cox regression model that included both transplantation and graft loss, compared with those remaining on dialysis, any kidney transplant provided survival benefit for wait-listed kidney transplant candidates regardless of diabetes as cause of kidney failure (HR 0.31 [95% CI 0.31, 0.32], *p*<0.001).

**Table 2:** Cox regression model of predictors for mortality rate for people with kidney failure (fully adjusted model with transplantation handled as a time varying covariate)

Variable	HR (95% CI)
Treatment	
Dialysis	REF
Transplant	0.26 (0.25, 0.27)
Age at wait-listing in years^a^	1.06 (1.05, 1.06)
Sex	
Female	REF
Male	1.08 (1.06, 1.11)
Ethnicity	
White	REF
Asian	0.89 (0.85, 0.92)
Black	0.85 (0.81, 0.90)
Other	0.69 (0.64, 0.76)
Unknown	1.06 (0.95, 1.18)
Cause of kidney failure	
Diabetes	REF
Glomerulonephritis	0.40 (0.38, 0.43)
Hypertension	0.45 (0.43, 0.48)
Other separate	0.42 (0.40, 0.43)
Polycystic kidney	0.35 (0.33, 0.36)
Pyelonephritis/reflux	0.49 (0.47, 0.52)
Unknown/missing	0.47 (0.45, 0.48)
Year of listing	0.93 (0.93, 0.93)

^a^Data are presented as HR (95% CI) for every 1 year increase in age

REF, reference

In a subset of data that included only people with kidney failure and diabetes, proceeding with kidney transplantation vs remaining on dialysis was associated with a survival benefit (HR 0.38 [95% CI 0.34, 0.42], *p*<0.001) (see ESM Table 4). Piecewise Cox models showed that this survival benefit is apparent immediately post-transplant vs remaining on dialysis: 0–30 days (HR 0.57 [95% CI 0.37, 0.86]); 31–180 days (HR 0.30 [95% CI 0.23, 0.39]); 181–365 days (HR 0.15 [95% CI 0.11, 0.20]); 1–3 years (HR 0.20 [95% CI 0.17, 0.23]); and 3–5 years (HR 0.27 [95% CI 0.21, 0.34]).

## Discussion

As the leading cause of kidney failure across many western countries, it is important that people with diabetes have the same opportunity to receive a kidney transplant if deemed clinically suitable for surgery. The primary findings of this population cohort study are as follows: (1) people with kidney failure and diabetes who are on the kidney transplant list are less likely to receive a transplant and more likely to remain wait-listed compared with other people with kidney failure; and (2) survival outcomes are better after kidney transplantation vs remaining on dialysis for people with kidney failure and diabetes. This analysis raises the question of whether people with kidney failure who have diabetes are disadvantaged by inequity of access for transplantation opportunities.

One explanation for our findings is that people with kidney failure and diabetes have additional medical comorbidities that skew their risk-vs-benefit ratio. For example, people with diabetes have been shown to have a much higher prevalence of peripheral arterial disease (PAD) [13], which could impact upon any post-transplant survival advantages. Registry analyses from the United States Renal Data System suggest that people with PAD who receive a deceased-donor kidney transplant have no survival advantage vs remaining on the transplant list (a survival advantage was only observed for recipients of living-donor kidneys) [14]. The lack of any survival advantage was determined to be due to a higher rate of post-transplant vascular events. Unfortunately, our data lacked information regarding medical or surgical comorbidities and the absence of such granular information is a limitation. However, registry analyses from Scotland [15], Spain [16, 17] and France [18] demonstrate that transplant recipients with a higher comorbidity burden have the greatest survival advantage after kidney transplantation and this is especially true in the context of underlying cardiovascular comorbidities. With CVD, one of the commonest medical comorbidities for people with kidney failure and diabetes [19, 20], competing medical comorbidity factors will influence perception of survival advantage after kidney transplantation. The critically important issue is that such discussions must be personalised to individual kidney transplant candidates for accurate risk-vs-benefit appraisal.

Another possibility is that people with kidney failure who have diabetes and are on the transplant list are subject to unfavourable bias from transplant professionals. While our analysis confirms that people with diabetes who are on the kidney transplant list have a survival advantage from kidney transplantation vs remaining on dialysis, we also demonstrate that they have an inferior survival rate, both in terms of graft loss and mortality risk, post-transplantation when compared with people without diabetes. This has also been shown in a population cohort analysis from Australia and New Zealand, where kidney transplant recipients with type 2 diabetes demonstrated 5 year mortality rates over two times higher than recipients without diabetes [4]. This may influence decision making when donor kidney offers are being considered, especially in the context of marginal donor kidneys, which may be deemed at higher risk (as per our findings that people with diabetes are disproportionably more likely to be transplanted with ECD kidneys). For example, one published study demonstrated that individuals who received kidneys from deceased donors with diabetes had a higher rate of all-cause allograft failure (HR 1.21 [95% CI 1.16, 1.26]) and death (HR 1.19 [95% CI 1.13, 1.24]) when compared with individuals who received a kidney from deceased donors without diabetes [21]. The worst individual and allograft survival outcomes were observed in kidney transplant recipients with diabetes who received deceased-donor kidneys from people with vs without diabetes. However, this is not a fair real-world comparison as kidney transplant candidates with diabetes are not offered a choice between multiple deceased-donor kidneys with or without diabetes. The critical question is whether wait-listed people with diabetes should accept a deceased-donor kidney with diabetes or wait for ‘better’ kidneys. That analysis has been performed in a different study, which compared survival benefits of kidney transplantation using deceased-donor kidneys with diabetes vs remaining on the waiting list in the USA between 1994 and 2015 [22]. Recipients of deceased-donor kidneys with diabetes displayed lower mortality risk compared with remaining on the waiting list and transplantation later with a deceased-donor kidney without diabetes (adjusted HR 0.91 [95% CI 0.84, 0.98]), with the greatest survival benefit attained by people with diabetes who had longer waiting times.

How can we advocate better for people with kidney failure who have diabetes? This analysis suggests there may be health inequalities that disadvantage people with kidney failure who have diabetes vs those with kidney failure from other causes. It is important to note that health inequalities have been previously noted among people with diabetes, especially on the basis of ethnicity [23, 24] or socioeconomic status [25], regarding access to optimal treatment. While we were able to adjust for ethnicity in our analysis, the lack of data regarding socioeconomic status meant we were unable to factor for this important variable in our analyses. Although post-transplant outcomes are inferior for people with kidney failure who have diabetes, they have improved significantly over time with enhanced post-transplant care [26], and commentators have argued that access to kidney transplantation must reflect this and be improved [27]. However, we must consider all potential barriers to kidney transplantation opportunities. While our analysis demonstrates the lower likelihood of people with kidney failure who have diabetes proceeding to transplantation after joining the transplant waiting list, we did not study whether there is inequity of access to join the waiting list. Many barriers have been identified regarding access to kidney transplantation opportunities including patient-related, physician/provider-related and/or system-related factors [28]. Acknowledging and understanding these barriers is important to help mitigate these disparities, which are likely to affect people with kidney failure who have diabetes more than others. Only then can we advocate for such people to ensure they get equal opportunities for the best available treatment tailored to their individual risk profile.

Our study benefits from being a contemporary analysis of a national population-based cohort. We used time-dependent Cox regression analysis, where exposure (dialysis vs transplantation) was handled as a time-dependent variable and is the approach most commonly used for studies in wait-listed kidney transplant candidates [2]. However, our study limitations must be appreciated for accurate interpretation of the data. First, we used an intention-to-treat approach that could bias results in favour of transplantation, as many people with kidney failure who are suspended or removed from the waiting list because of ill health have an increased mortality risk [29]. However, granular data with regards to reasons for suspension or removal are not available for access. Post-transplantation diabetes is a common medical complication associated with solid organ transplantation and can influence patient or graft survival [30]; however, incidence rates are not recorded in national registries and therefore we could not account for this in our analysis. We also lack data with regards to type or duration of diabetes, which is not recorded in the UK Transplant Registry. Our dataset comprised of people on dialysis only awaiting their first kidney transplant and therefore provides no evidence in the setting of advanced chronic kidney disease or a failed kidney transplant. Furthermore, we explored access to transplantation opportunities after wait-listing and post-transplant outcomes; our study was not designed to explore whether people with kidney failure who have diabetes have an equal opportunity to access the waiting list itself (referral and/or assessment barriers). As mentioned earlier in the discussion, lack of medical comorbidity and socioeconomic data limits interpretation of survival probabilities in the setting of specific health and social burdens, tipping the balance in borderline risk-vs-benefit calculations. We believe further work in this area is warranted and requires this additional information for facilitation of nuanced risk-vs-benefit decision making. Finally, this analysis has focused solely upon survival benefits associated with transplant surgery for people with kidney failure with diabetes and does not address quality of life benefits which are beyond the scope of this study.

To conclude, in this contemporary national cohort study of people with kidney failure listed for transplantation, proceeding with transplant surgery affords a survival advantage to wait-listed kidney transplant candidates with diabetes. However, the data suggest that people with kidney failure with diabetes have reduced opportunities to proceed with transplantation, possibly reflecting a selection bias that works against people with kidney failure with diabetes and/or the presence of concomitant medical comorbidities that we could not explore in detail. Although survival benefits at a population-level must be translated to individual kidney transplant candidates with personalised risk counselling, our data suggest that transplantation remains the treatment of choice for all suitable people with kidney failure and must be considered the default choice even in the context of diabetes unless specifically contraindicated.

## Supplementary Information

Below is the link to the electronic supplementary material.

ESM (PDF 203 KB)

## Data Availability

The data used for this analysis is available upon request from NHS Blood & Transplantation.

## Abbreviations

CIT: Cold ischaemia time
cRF: Calculated reaction frequency
ECD: Expanded-criteria donor
NHSBT: National Health Service Blood and Transplant
PAD: Peripheral arterial disease
SCD: Standard-criteria donor
