# Supplementary material for: Mortality risk for kidney transplant candidates with diabetes: a population cohort study
Source: Diabetologia. 2024 Aug 6;67(11):2530–8. doi: 10.1007/s00125-024-06245-x (PMC11519178; doi:10.1007/s00125-024-06245-x)
Supplement: Supplementary file 1 — ESM (PDF 203 KB) [file 125_2024_6245_MOESM1_ESM.pdf]

Supplementary Figure 1. Kidney replacement therapy counts for kidney failure people with versus without diabetes (Tx = transplantation, No Tx = dialysis)

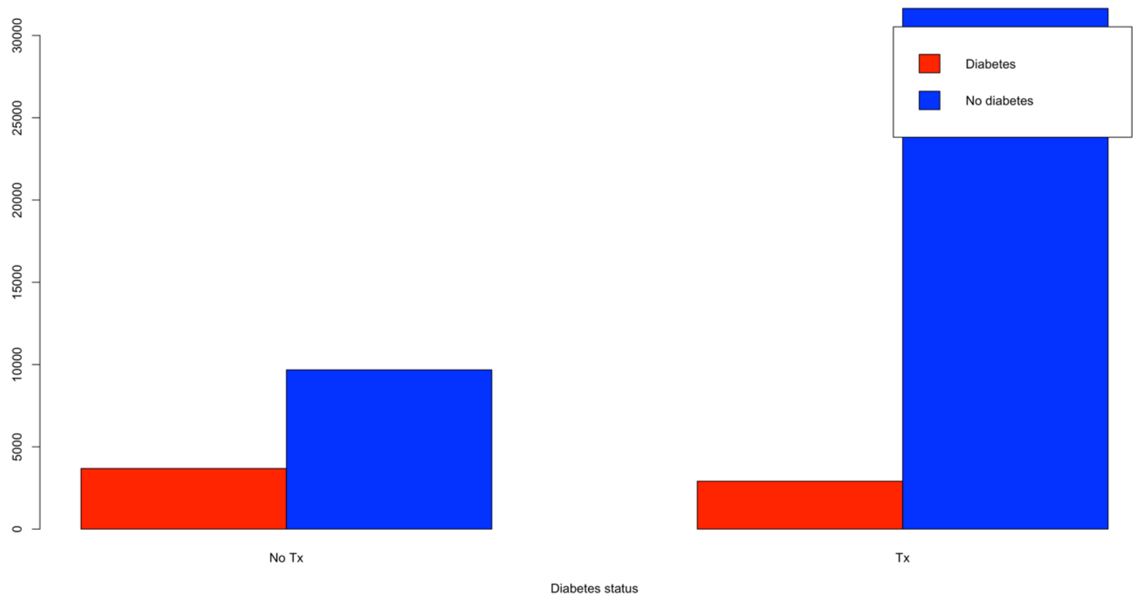

Supplementary Figure 2. Kidney replacement therapy counts for kidney failure people with versus without diabetes (SCD = transplantation with standard criteria donor kidney, LD = transplantation with living donor kidney, ECD = transplantation with expanded criteria donor kidney)

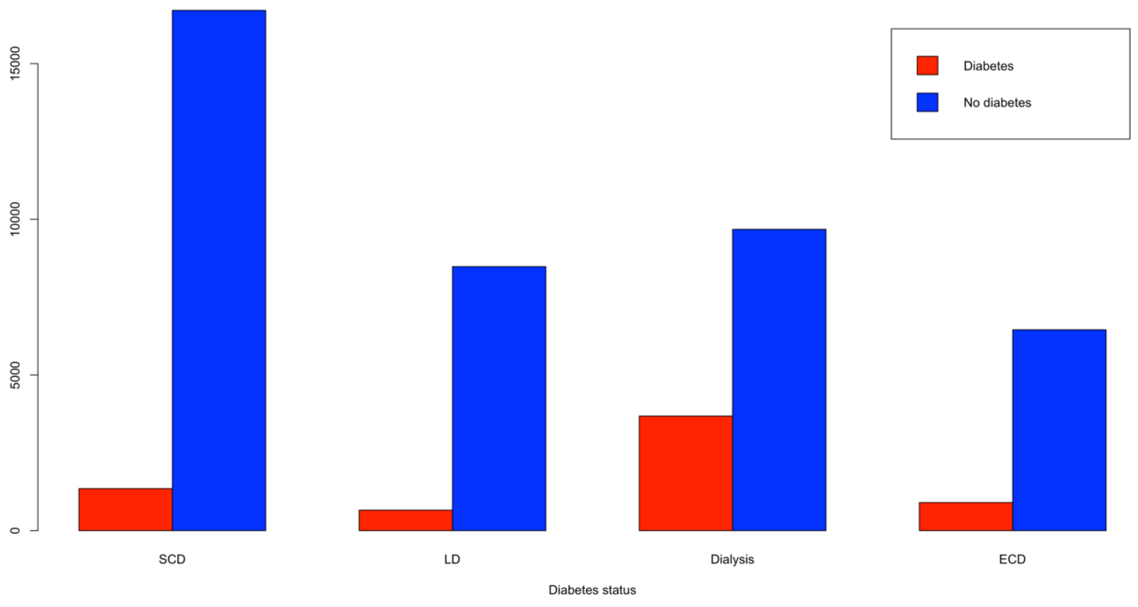

Supplementary Table 1. Baseline demographic comparison of kidney failure people wait-listed on the kidney transplant list stratified by diabetes status

Characteristic		Diabetes	No diabetes
**N (%)**		6,594 (13·8%)	41,323 (86·2%)
**Transplant list status**	Transplant	2,913 (44·2%)	31,645 (76·6%)
	Dialysis	3,681 (55·8%)	9,678 (23·4%)
**Sex**	Female	2,253 (34·2%)	15,865 (38·4%)
	Male	4,341 (65·8%)	25,458 (61·6%)
**Age at listing (years, SD)**		50·5, 12·0	47·5, 14·3
**Ethnic Group**	White	4,559 (69·1%)	31,680 (76·7%)
	Asian	1,274 (19·3%)	5,014 (12·1%)
	Black	492 (7·5%)	3,079 (7·5%)
	Other	219 (3·3%)	1,164 (2·8%)
	Unknown	50 (0·8%)	386 (0·9%)
**Time on transplant list (days, SD)**		771, 622	916, 841

SD – standard deviation

Supplementary Table 2. Baseline demographic and immunological comparison of kidney transplant recipients stratified by diabetes status

Characteristic		Diabetes	No diabetes
**N (%)**		2,913 (8·4%)	31,645 (91·6%)
**Sex**	Female	901 (30·9%)	12,001 (37·9%)
	Male	2,012 (69·1%)	19,644 (62·1%)
**Age at listing (years, SD)**		52·5, 11·2	45·8, 14·0
**Age at transplant (years, SD)**		54·4, 11·3	48·1, 14·1
**Ethnic Group**	White	1,919 (65·9%)	24,756 (78·2%)
	Asian	642 (22·0%)	3,574 (11·3%)
	Black	223 (7·7%)	2,150 (6·8%)
	Other	111 (3·8%)	856 (2·7%)
	Unknown	18 (0·6%)	309 (1·0%)
**Time on transplant list (days, SD)**		724, 630	862, 843
**HLA group**	Level 1	319 (11·0%)	3,813 (12·1%)
	Level 2	739 (25·4%)	9,828 (31·1%)
	Level 3	1,428 (49·0%)	14,016 (44·3%)
	Level 4	427 (14·7%)	3,984 (12·6%)
**Cold ischaemic time (hours, SD)**		13·1, 7·2	12·9, 7·8
**Calculated reaction frequency (mean)**		13%	19%

SD – standard deviation

Supplementary Table 3. Cox regression model of predictors for graft loss after kidney transplantation

Variable		Hazard Ratio (95% CI)
Cause of kidney failure	Other causes	REF
	Diabetes	1·36 (1·20-1·53)
Median Age at waitlisting in years (IQR)		1·00 (0·99-1·00)
Sex	Female	REF
	Male	1·08 (0·94-1·24)
Ethnicity	White	REF
	Asian	1·66 (0·99-2·78)
	Black	1·78 (1·49-2·12)
	Other	1·29 (1·01-1·65)
	Unknown	0·36 (0·16-0·81)
Waiting time		0·99 (0·98-1·00)
HLA mismatch	Level 1	REF
	Level 2	1·14 (0·87-1·48)
	Level 3	1·20 (0·94-1·54)
	Level 4	1·27 (0·99-1·63)
Cold ischaemic time		1·01 (1·00-1·02)
Calculated reaction frequency		1·00 (1·00-1·00)

Supplementary Table 4. Cox regression model of predictors for mortality for kidney failure patients with diabetes only (fully adjusted model with transplantation handled as a time varying covariate)

Variable		Hazard Ratio (95% CI)
**Treatment**	Dialysis	REF
	Transplant	0·38 (0·34-0·42)
**Median Age at waitlisting in years (IQR)**		1·05 (1·04-1·05)
**Sex**	Female	REF
	Male	0·94 (0·87-1·02)
**Ethnicity**	White	REF
	Asian	0·78 (0·71-0·85)
	Black	0·68 (0·59-0·80)
	Other	0·76 (0·61-0·95)
	Unknown	1·65 (0·91-2·99)
**Year of listing**		0·92 (0·91-0·93)
